# Supplementary material for: Clipper: p-value-free FDR control on high-throughput data from two conditions
Source: Genome Biol. 2021 Oct 11;22:288. doi: 10.1186/s13059-021-02506-9 (PMC8504070; doi:10.1186/s13059-021-02506-9)
Supplement: Supplementary file 3 — Additional file 3 Review history [file 13059_2021_2506_MOESM3_ESM.docx]

Review History

**First round of review**

**Reviewer 1**

**Were you able to assess all statistics in the manuscript, including the appropriateness of statistical tests used?**

Yes: All tests appropriate.

**Were you able to directly test the methods?**

No

**Comments to author:**

In this manuscript, authors develop a framework named Clipper that adopts the foundational Barber-Candes (BC) procedure for false discovery rate control without the p-values in high dimensional enrichment and differential analysis settings. The BC procedure does not require well-calibrated p-values and hence is applicable to a wide variety of settings. The BC procedure is developed for variable selection in a linear model and Clipper adopts this idea for identifying enriched features in experiment vs. background settings and differential features across two experimental conditions with genomic applications in mind. After evaluating Clipper in a number of generic simulation scenarios, four widely encountered genomic applications (peak finding in ChIP-seq, peptide identification from mass spectrometry data, differential expression analysis in RNA-seq, differential interaction analysis in Hi-C) are considered. Overall, Clipper shows good control of FDR and can provide power advantages compared to generic FDR control procedures. The authors perform a thorough theoretical analysis of how the two summary statistics (contrast scores) that Clipper leverages provide FDR control.

While I think the general framework has a lot of potential because FDR control in high throughput experiments with small numbers of replicates is challenging, there are a number of significant issues that were not addressed. First, the notion of discrete lists from FDR control is never quite the final/key result of interest for a high throughput experiment. The experiments presented should really also compare the ranking of the interesting features from Clipper vs. alternatives or present a useful ranking scheme for Clipper. It would also be really useful to have explicit connections to the BC knockoff construction, which the authors achieve by contrast scores. Below are some more detailed comments on the major issues.

1) Given the small number of replicates in simulations, it is essential to consider criteria beyond FDR control and power. In particular, it is essential to (i) more explicitly illustrate how Clipper is achieving good FDR control and power – why are the presented contrast scores that are not taking into account within group variations good choices?; (ii) investigate whether the ranking of Clipper results (with its contrast scores) are in agreement with other ranking criteria. For example, in differential expression (DE) analysis, while the resulting p-values of DEseq2 may not be calibrated due to model misspecification, the actual test statistics from the hierarchical model that it employs perform well in terms of ranking the genes. Hence, we would expect DEseq2 to generate low empirical FDR when comparing top DE genes.

When the number of replicates is this small, these genomic studies are more about getting a reliable ranking and also reproducibility (e.g., with IDR in ChIP-seq

experiments) than getting a discrete list of interesting features. In general, I found the Clipper contrast scores a bit unconventional since they are really simple summaries of the data and do not perform any “information borrowing” which is shown to be especially powerful in the DE analysis setting.

1.1) In the generic simulations, the set of interesting features are only 10% of the total numbers of features. This parameter should be varied to establish robustness.

2) In general, the design of the omics computational experiments needs more attention so that they are realistic and are covering general scenarios. In the current form, these experiments are not representative of actual datasets.

2.1)	ChIP-seq data with synthetic spike ins.

In the ChIP-seq simulations, 25 peaks identified from an actual analysis of one ChIP and a control sample are inserted into a control sample as spike-ins. The authors focus on chromosome 1 and insert one peak from these 25 into promoters of genes. The statement “For each promoter region, we selected one of the 25 true peaks whose length was closest to the length of the promoter region..” does not make much sense because promoter is defined as 1000/2000bp upstream of TSS, so each gene promoter should have the same length unless the authors are using a different definition.

The authors only cover the one ChIP vs. one control replicate setting. The standard today is at least 2 ChIP replicates.

The way that the authors computationally simulated the data is somewhat an outdated way of generating simulated data. There are now several ChIP-seq simulators (e.g., ChIPulate, isChIP, simChIP) that should enable the authors consider a broader and more realistic set of scenarios and also account for “effect sizes/strengths of the peaks” etc.

2.2)	For MS data generation, the readers are referred to a future manuscript and it is

not possible to evaluate how realistic these experiments are. For the purposes of the review process, this information should have been included.

2.3)	Bulk RNA-seq data spike-ins. While the experiments of the authors are not

unreasonable, it would be much more impactful, if the authors took the computational experiments from the source DEseq2 or edgeR papers. In fact, Gierlinski et al., Bioinformatics, 2015, has a large set of replicate RNA-seq experiments that can also be leveraged for a more realistic setting. Korthauer et al., Genome Biology, 2019 does a good job of generating realistic computational experiment that authors may consider.

2.4)	Hi-C data. While the Hi-C data generation with synthetic spike-ins take into

account the genomic distance effect between region pairs, it does not take into account the spatial correlation between the regions.

3.	The theoretical calculations for FDR control rely on having the signs of contrast scores of uninteresting features to be equally likely positive or negative (e.g., Lemma 1). This is an assumption that will be violated for enrichment analysis in ChIP-seq data as the background of the ChIP sample is different than the “input” control by construction. This will lead to input to be generally (normalization also has a role to play here) slightly larger than the ChIP background counterpart. It is possible that the theoretical result will hold with a

modification, but as I have indicated in #2, application to genomic problems require more investigation.

4.	Finally, if the RNA-seq analysis focused on differential expression in scRNA-seq (in addition

to bulk), the relevance of the paper for the genomics community would be higher.

**Reviewer 2**

**Were you able to assess all statistics in the manuscript, including the appropriateness of statistical tests used?**

Yes: As this is a statistical method, the review itself below is a review of the statistical methods.

**Were you able to directly test the methods?**

Yes

**Comments to author:**

This is an interesting, potentially impactful, contribution to an important aspect of genomic data analysis. The authors describe new methodology to more faithfully control for False Discovery Rate than currently established methodology. The proposed methodology adapts previous work on selection procedures from Candes to testing situations applicable across multiple genomic measurements. Overall, the metodological contribution is an innovative application and is well characterized formally (using proof mechanisms as established in the Candes line of work). The empirical characterization of the method as provided here can be improved.

- Contrast Scores. The statistics on which this method relies are contrast scores, of which the authors present two, one based on differences between empirical means of two groups (diff), and one based on a signed maximum absolute value of means of two groups (enrich). I find the introduction of the enrich contrast score, confusing and perhaps unnecessary. As far as I could understand, the latter is mainly applicable in the enrichment testing case (average of one group is strictly greater than the other group), but the diff contrast score is also applicable in that case. And it seems that empirically the diff contrast score has better performance as well. That is, there is no evidence, as far as I could tell, of the enrichment contrast score providing a practical or theoretical advantage. I would recommend

(a) providing evidence of this practical advantage, or simplify the presentation of the paper by considering only the diff contrast score.

(b) guidelines on specific situations when each kind of contrast score is appropriate. For instance when testing the Clipper R tool and using the DEG example, the "analysis" parameter is set to "differential" but the contrast score used by default is "max", which I found surprising. In general, I find the whole need for this second type of contrast score confusing

- Applicability beyond two-group testing. The limitation of this procedure to two-group testing is a severe handicap. In a majority of analysis we tend to see the need to include additional terms in, e.g., linear regression models to control for technical or biological sources of variability beyond the direct effect being tested. Given that the original application from Candes et al. was selection in linear models, wouldn't using parameter estimates for appropriately setup up covariates (related to the diff contrast score) as contrast scores work here as well? The authors should comment on this on the manuscript, if extending the method to this is beyond scope of the paper.

- Simulations. I am not sure the simulations are designed to be the most informative. I am concerned that the bulk of the simulations are focused on cases with very small number of replicates (1 v. 1, 3 v. 3). This is becoming increasingly uncommon (perhaps not in the proteomics and hi-c use cases?) and would prefer that simulations are refocused to concentrate on what may be more important characteristics:

(a) behavior for moderate sample sizes (this is included as 10v10 testing but not highlighted, I suspect because the difference in performance to other methods is not as significant),

(b) spends more time discussing how the performance of these methods degrade as the independence assumption across tests is violated (this is a pervasive problem in genomic analysis and it is cursorily addressed in the current set of simulations. In particular, there is no case where correlated tests are used in Poisson or Negative Binomial tests, only on the Normal case.

(c) In many of the plots there is no distincation made of the data generating distribution (between Normal, Poisson or NB), and is hard to understand behavior of the methods.

(d) the simulations concentrate on situations where variability across tests is fairly stable whereas (for instance in the NB case all features have the same overdispersion parameter). This is not reflective of many genomic measurements where there is a fair amount of differences in variation across features.

- differential expression analysis. I am sincerely surprised that there is such a difference in behavior between DESeq2 and EdgeR in this analysis. It would be good to better understand how these methods were applied, for instance, I would expect that the regularization method approach in DESeq2 would result in lower number of significant results. I did not see specifics of how these methods were used in the paper. Similarily, in my experience for common RNA-seq datasets using importance hypothesis weighting tends to have moderate to substantial effects, yet I am surprised that it had no effect in this case. I would suggest, (a) that the authors provide more detail on how these methods were used, and that (b) an additional analysis is included, ideally using an established benchmark dataset.

**Authors Response**

**Point-by-point responses to the reviewers’ comments:**

*We thank the two reviewers for their constructive comments. We have addressed all of them and modified the paper accordingly. Our detailed point-by-point answers are in the following pages.*

Reviewer 1

In this manuscript, authors develop a framework named Clipper that adopts the foundational Barber-Candes (BC) procedure for false discovery rate control without the p-values in high dimensional enrichment and differential analysis settings. The BC procedure does not require well-calibrated p-values and hence is applicable to a wide variety of settings. The BC procedure is developed for variable selection in a linear model and Clipper adopts this idea for identifying enriched features in experiment vs. background settings and differential features across two experimental conditions with genomic applications in mind. After evaluating Clipper in a number of generic simulation scenarios, four widely encountered genomic applications (peak finding in ChIP-seq, peptide identification from mass spectrometry data, differential expression analysis in RNA-seq, differential interaction analysis in Hi-C) are considered. Overall, Clipper shows good control of FDR and can provide power advantages compared to generic FDR control procedures. The authors perform a thorough theoretical analysis of how the two summary statistics (contrast scores) that Clipper leverages provide FDR control. While I think the general framework has a lot of potential because FDR control in high throughput experiments with small numbers of replicates is challenging, there are a number of significant issues that were not addressed.

*We thank the reviewer for appreciating the merit of our work, the clear summary above, and the constructive suggestions below.*

First, the notion of discrete lists from FDR control is never quite the final/key result of interest for a high throughput experiment. The experiments presented should really also compare the ranking of the interesting features from Clipper vs. alternatives or present a useful ranking scheme for Clipper.

*We have addressed this issue in our revised manuscript. Please see our detailed responses to the reviewer’s major comment R1.1 below.*

It would also be really useful to have explicit connections to the BC knockoff construction, which the authors achieve by contrast scores. Below are some more detailed comments on the major issues.

*We have added a paragraph to the Discussion section about the relevance and distinction between Clipper and the BC knockoff construction. Here we paste it below.*

*“Clipper’s FDR control procedures (BC and GZ procedures in Methods) are motivated by the Barber-Candès (BC)’s knockoff paper [1] and the Gimenez-Zou’s multiple knockoff paper [2], but we do not need to construct knockoffs in enrichment analysis when two conditions have the same number of replicates; the reason is that the replicates under the background condition serve as natural negative controls. For differential analysis and enrichment analysis with unequal numbers of replicates, in order to guarantee the theoretical assumptions for FDR control, Clipper uses per-mutations instead of the complicated knockoff construction because Clipper only examines features marginally and does not concern about features’ joint distribution.”*

Here are some major comments for the authors:

Comment R1.1 Given the small number of replicates in simulations, it is essential to consider criteria beyond FDR control and power. In particular, it is essential to (i) more explicitly illustrate how Clipper is achieving good FDR control and power – why are the presented contrast scores that are not taking into account within group variations good choices?; (ii) investigate whether the ranking of Clipper results (with its contrast scores) are in agreement with other ranking criteria. For example, in differential expression (DE) analysis, while the resulting p-values of DEseq2 may not be calibrated due to model misspecification, the actual test statistics from the hierarchical model that it employs perform well in terms of ranking the genes. Hence, we would expect DEseq2 to generate low empirical FDR when comparing top DE genes. When the number of replicates is this small, these genomic studies are more about getting a reliable ranking and also reproducibility (e.g., with IDR in ChIP-seq experiments) than getting a discrete list of interesting features. In general, I found the Clipper contrast scores a bit unconventional since they are really simple summaries of the data and do not perform any “information borrowing” which is shown to be especially powerful in the DE analysis setting.

*Answer to R1.1 We thank the reviewer for these detailed, valuable suggestions. We have followed the reviewer’s suggestions and added the following analyses and explanations:*

*1. We have added two simulation designs to compare the default Clipper with a Clipper variant that uses the t statistic, which takes into account within-condition/group variations. In detail, the t statistic is used as an alternative contrast score in the 3vs3 enrichment analysis design; it is used as a summary statistic to calculate the degree of interestingness in the 3vs3 differential analysis design. To explain how we use the t statistic as a summary statistic, and to highlight that Clipper allows alternative summary statistics, we have added the following sentences to the Methods section:*

*“Notably, other summary statistics can also be used to construct contrast scores. For example, an alternative summary statistic is the t statistic from the two-sample t test:*

*” We have also added the definition of the Clipper variant using the above t statistic to Supp. Section S2 “The Clipper methodology.”*

*In our newly added Supp. Figs. S13–S14 (pasted below for the reviewers’ convenience), the comparison results show that the default Clipper achieves higher power compared to the Clipper t statistic variant. Below is our explanation of this phenomenon, which has been added as a paragraph to the Discussion section.*

*“An advantage of Clipper is that it allows other definitions of contrast scores, such as the two-sample t statistic that considers within-condition variances. Empirical evidence (Supp. Figs. S13 and S14) shows that the Clipper variant using the two-sample t statistic is under¬powered by the default Clipper, which uses the minus summary statistic (difference of two conditions’ sample means) as the contrast score in the 3vs3 enrichment analysis or as the degree of interestingness in the 3vs3 differential analysis (see Methods). Here is our current interpretation of this seemingly counter-intuitive result.*

*·	First, both the minus statistic and the t statistic satisfy Clipper’s theoretical conditions (Lemmas 1 and 3 in Supp. Section S2), which guarantee the FDR control by the BC and GZ procedures; this is confirmed in Supp. Figs. S13 and S14. Hence, from the FDR control perspective, Clipper does not require the adjustment for within-condition variances by using a t statistic.*

*·	Second, Clipper is different from the two-sample t test or the regression-based t test, where the t statistic was purposely derived as a pivotal statistic so that its null distribu¬tion (the t distribution) does not depend on unknown parameters. Since Clipper does not require a null distribution for each feature, the advantage of the t statistic being pivotal no longer matters.*

*·	Third, the minus statistic only requires estimates of two conditions’ mean parameters, while the t statistic additionally requires estimates of the two conditions’ variances. Hence, when the sample sizes (i.e., the numbers of replicates) are small, the two more parameters that need estimation in the t statistic might contribute to the observed power loss of the Clipper t statistic variant. Indeed, the power difference between the two statistics diminishes as the sample sizes increase from 3vs3 in Supp. Figs. S13–S14 to 10vs10 in Supp. Fig. S10 (where we compared the default Clipper with BH-pair- parametric, which is based on the two-sample t test and is highly similar to the Clipper t statistic variant).*

*·	Fourth, we observe empirically that a contrast score would have better power if its distribution (based on its values of all features) has a larger range and a heavier right tail (in the positive domain). Compared to the minus statistic, the t statistic has a smaller range and a lighter right tail due to its adjustment for features’ within-condition variances (Supp. Fig. S28). This observation is consistent with the power difference of the two statistics.*

*Beyond our current interpretation, however, we admit that future studies are needed to explore alternative contrast scores and their power with respect to data characteristics and analysis tasks. Furthermore, we may generalize Clipper to be robust against sample batch effects by constructing the contrast score as a regression-based test statistic that has batch effects removed.”*

*Figure S13: In the 3vs3 enrichment analysis with and without outliers, comparison of the default Clipper, the Clipper variant using the t statistic as the contrast score (Clipper-t), and six generic FDR control methods (Clipper BH-pooled, BH-pair-correct, BH-pair-2as1, BH-pair-mis, locfdr-emp, and locfdr-swap) in terms of their FDR control and power. At target FDR thresholds q ∈1%, 2%,--- , 10%, each method’s actual FDRs and power are evaluated on 200 simulated datasets with d = 10,000 features generated from the Gaussian distribution, the Poisson distribution, or the negative binomial distribution under homogeneous (columns 1 and 2) and heterogeneous (columns 3 and 4) background scenarios. Clipper achieves higher power than Clipper-t does.*

*Figure S14: In the 3vs3 differential analysis with and without outliers, comparison of the default Clipper, the Clipper variant using the t statistic to calculate the degree of interestingness (Clipper-t), and six generic FDR control methods (Clipper BH-pooled, BH-pair-correct, BH-pair-2as1, BH-pair-mis, locfdr-emp, and locfdr-swap) in terms of their FDR control and power. At target FDR thresholds q ∈1%, 2%,--- , 10%, each method’s actual FDRs and power are evaluated on 200 simulated datasets with d = 10,000 features generated from the Gaussian distribution, the Poisson distribution, or the negative binomial distribution under homogeneous (columns 1 and 2) and heterogeneous (columns 3 and 4) background scenarios. Clipper achieves higher power than Clipper-t does.*

*Figure S28: In the 3vs3 enrichment analysis, distributions of contrast scores used by two Clipper variants: the default Clipper using the minus contrast score (top) and the Clipper variant using the two-sample statistic (bottom). Features are generated from the Gaussian distribution under the hetero-geneous background scenario (see Supp. Section S4). The vertical dashed lines indicate the contrast score cutoffs found by the BC procedure at the target FDR threshold = 1%. The distribution of the minus contrast scores has a heavier right tail (522%) than that of the distribution of the statistic contrast scores (119%).*

*2. Regarding the “information borrowing” issue raised by the reviewer, we would like to clarify that, although the contrast score construction for each feature does not use information of other features, the contrast score thresholding step does use information of all features; this phenomenon is known as the “power of multiple testing.” We would also like to clarify that other contrast scores that borrow information from other features, as long as they satisfy the conditions in Lemmas 1 and 3, can also be used in Clipper. We have added the following sentences to the Discussion section for these two clarifications.*

*“Notably, Clipper achieves FDR control and high power even using simple contrast scores, which are calculated for individual features without borrowing information from other fea¬tures. However, Clipper does leverage the power of multiple testing by setting a contrast score threshold based on all features’ contrast scores. This is a likely reason why Clipper achieves good power even with simple contrast scores.”*

*3. We totally agree that the ranking and reproducibility of the discoveries are important in real data analysis. Hence, we have added a new analysis about the ranking and reproducibility of differentially expressed genes (DEGs) identified from bulk RNA-seq data. The analysis results are added as two paragraphs to the Section “DEG identification from bulk RNA-seq data (differential analysis I)”, as well as in Fig. 3 and Supp. Figs. S15–S17. For the reviewer’s convenience, below we have pasted the added paragraphs and figures.*

*“In addition, we compared the DEG ranking by Clipper, edgeR, and DESeq2 in two ways. First, for true DEGs, we compared their ranking by each method with their true ranking based on true expression fold changes (from large to small, as in semi-synthetic data gen¬eration in Supp. Section S6.3). Specifically, we ranked true DEGs using Clipper’s contrast scores (from large to small), edgeR’s p-values (from small to large), or DESeq2’s p-values (from small to large). Our results in Fig. 3b and Supp. Figs. S15b–S17b show that Clip-per’s contrast scores exhibit the most consistent ranking with the ranking based on true fold changes. Second, to compare the power of Clipper, edgeR, and DESeq2 based on their DEG rankings instead of nominal p-values, we calculated their power under the actual FDRs, which only depend on gene rankings (for the definition of actual FDR, see Supp. Section S6.3). Fig. 3a and Supp. Figs. S15a–S17a show that, when Clipper, edgeR, and DESeq2 have the same actual FDR, Clipper consistently outperforms edgeR and DESeq2 in terms of power, i.e., Clipper has the most true DEGs in its top ranked genes.*

*We also compared the reproducibility of Clipper, edgeR, and DESeq2 in the presence of sam-pling randomness. Specifically, we used two semi-synthetic datasets (generated independently from the same procedure in Supp. Section S6.3) as technical replicates and computed Clip-per’s contrast scores and edgeR’s and DESeq’s p-values on each dataset. For each method, we evaluated its reproducibility between the two semi-synthetic datasets by computing three criteria—the irreproducibility discovery rate (IDR) [3], Pearson correlation, and Spearman correlation—using its contrast scores or negative log10 transformed p-values. Fig. 3c and Supp. Figs. S15–S17c show that Clipper’s contrast scores have higher reproducibility by all three criteria compared to edgeR’s and DESeq2’s p-values.”*

*Figure S15: Comparison of Clipper and two popular DEG identification methods—edgeR and DESeq2—in DEG analysis on semi-synthetic bulk RNA-seq data (generated from hu¬man monocyte real data using simulation strategy 1 in Supp. Section S6.3). (a) FDR control, power given the same target FDR, and power given the same actual FDR. (b) Ranking consistency of the true DEGs among the top 100 DEGs identified by each method. The consistency is defined between the genes’ ranking based on edgeR/DESeq2’s p-values or Clipper’s contrast scores and their ranking based on true expression fold changes. (c) Reproducibility between two semi-synthetic datasets as technical replicates. Three reproducibility criteria are used: the IDR, Pearson correlation, and Spearman correala-tion. Each criterion is calculated for edgeR/DESeq2’s p-values or Clipper’s contrast scores on the two semi-synthetic datasets. Among the three methods, only Clipper controls the FDR, and Clipper achieves the highest power, the best gene ranking consistency, and the best reproducibility.*

*Figure S16: Comparison of Clipper and two popular DEG identification methods—edgeR and DESeq2—in DEG analysis on semi-synthetic bulk RNA-seq data (generated from yeast real data using simulation strategy 1 in Supp. Section S6.3). (a) FDR control, power given the same target FDR, and power given the same actual FDR. (b) Ranking consistency of the true DEGs among the top 100 DEGs identified by each method. The consistency is defined between the genes’ ranking based on edgeR/DESeq2’s p-values or Clipper’s contrast scores and their ranking based on true expression fold changes. (c) Reproducibility between two semi-synthetic datasets as technical replicates. Three reproducibility criteria are used: the IDR, Pearson correlation, and Spearman correalation. Each criterion is calculated for edgeR/DESeq2’s p-values or Clipper’s contrast scores on the two semi-synthetic datasets. Among the three methods, only Clipper controls the FDR, and Clipper achieves the highest power, the best gene ranking consistency, and the best reproducibility.*

*Figure S17: Comparison of Clipper and two popular DEG identification methods—edgeR and DESeq2—in DEG analysis on semi-synthetic bulk RNA-seq data (generated from yeast real data using simulation strategy 2 in Supp. Section S6.3). (a) FDR control, power given the same target FDR, and power given the same actual FDR. (b) Ranking consistency of the true DEGs among the top 100 DEGs identified by each method. The consistency is defined between the genes’ ranking based on edgeR/DESeq2’s p-values or Clipper’s contrast scores and their ranking based on true expression fold changes. (c) Reproducibility between two semi-synthetic datasets as technical replicates. Three reproducibility criteria are used: the IDR, Pearson correlation, and Spearman correalation. Each criterion is calculated for edgeR/DESeq2’s p-values or Clipper’s contrast scores on the two semi-synthetic datasets. Among the three methods, only Clipper controls the FDR, and Clipper achieves the highest power, the best gene ranking consistency, and the best reproducibility.*

Comment R1.2 In the generic simulations, the set of interesting features are only 10% of the total numbers of features. This parameter should be varied to establish robustness.

*Answer to R1.2 We thank the reviewer for this suggestion. Following this, we have added two more simulation settings, where 20% and 40% of the features are set to be interesting. We have added Supp. Fig. S12 and sentences to the Results section (Page 5 of the revised manuscript). For reviewer’s convenience, below we have pasted the added figure and text.*

*“In addition, for the 3vs3 enrichment analysis, we also varied the proportion of interesting features as 10%, 20%, and 40%. The comparison results in Supp. Fig. S3 (columns 1 and 3 for 10%) and Supp. Fig. S12 (for 20% and 40%) show that the performance of Clipper is robust to the proportion of interesting features.”*

Comment R1.3 In general, the design of the omics computational experiments needs more at-tention so that they are realistic and are covering general scenarios. In the current form, these experiments are not representative of actual datasets.

·	ChIP-seq data with semi-synthetic spike ins. In the ChIP-seq simulations, 25 peaks identified from an actual analysis of one ChIP and a control sample are inserted into a control sample as spike-ins. The authors focus on chromosome 1 and insert one peak from these 25 into promoters of genes. The statement “For each promoter region, we selected one of the 25 true peaks whose length was closest to the length of the promoter region..” does not make much sense because promoter is defined as 1000/2000bp upstream of TSS, so each gene promoter should have the same length unless the authors are using a different definition. The authors only cover the one ChIP vs. one control replicate setting. The standard today is at least 2 ChIP replicates. The way that the authors computationally simulated the data is somewhat an outdated way of generating simulated data. There are now several ChIP-seq simulators (e.g., ChIPulate, isChIP, simChIP) that should enable the authors consider a broader and more realistic set of scenarios and also account for “effect sizes/strengths of the peaks” etc.

·	For MS data generation, the readers are referred to a future manuscript and it is not possible to evaluate how realistic these experiments are. For the purposes of the review process, this information should have been included.

·	Bulk RNA-seq data spike-ins. While the experiments of the authors are not unreasonable, it would be much more impactful, if the authors took the computational experiments from the source DEseq2 or edgeR papers. In fact, Gierlinski et al., Bioinformatics, 2015, has a large set of replicate RNA-seq experiments that can also be leveraged for a more realistic setting. Korthauer et al., Genome Biology, 2019 does a good job of generating realistic computational experiment that authors may consider.

·	Hi-C data. While the Hi-C data generation with synthetic spike-ins take into account the genomic distance effect between region pairs, it does not take into account the spatial corre-lation between the regions.

*Answer to R1.3 We thank the reviewer for the detailed suggestions. We have followed the suggestions and modified our omics data analysis.*

*·	For peak calling from ChIP-seq data, we have changed our synthetic data generation pro-cedures by using ChIPulate, a simulator suggested by the reviewer, to generate synthetic*

*Figure 4: Comparison of Clipper and popular bioinformatics methods in terms of FDR control and power. (a) peaking calling analysis on semi-synthetic ChIP-seq data; (b) peptide identi¬fication on real proteomics data; (c) DEG analysis on synthetic 10x Genomics scRNA-seq data; (d) DIR analysis on semi-synthetic Hi-C data. In all four panels, the target FDR threshold ranges from 1% to 10%. In the “Actual FDR vs. Target FDR” plot of each panel, points above the dashed diagonal line indicate failed FDR control; when this happens, the power of the corresponding methods is not shown, including HOMER in (a), MACS2 for target FDR less than 5% in (a), edgeR in (c), and multiHICcom-pare, and FIND in (d). In all four applications, Clipper controls the FDR while maintaining high power, demonstrating Clipper’s broad applicability in high-throughput data analyses.*

*·	For MS data, we have added the following text, which explains how we generated the benchmark dataset for peptide identification, to Supp. Section S6.2.*

*“We purchased the complex proteomics standard (CPS) (part number 400510) from Agilent (Agilent, Santa Clara, CA, USA). The CPS contains soluble proteins extracted from the archaeon Pyrococcus furiosus (Pfu), which has a complete protein database; that is, all proteins from Pfu were catalogued into its protein database with known protein sequences. We subjected the CPS to a shotgun proteomics analysis that generated mass spectra of Pfu.*

*To generate a benchmark dataset, we first generated a reference database by concatenating the Uniprot Pyrococcus furiosus (Pfu) database, the Uniprot Human database, and two contaminant databases: the CRAPome [4] and the contaminant database from MaxQuant [5]. During the process, we purified the reference database by first performing in silico digestion of Pfu proteins and then removing human proteins that contained Pfu peptides from the reference database. We then input the Pfu mass spectra (from the CPS) and the purified reference database into Mascot. We considered a target PSM as true if Mascot reported its protein as from Pfu or the two contaminants; otherwise (if from Human), we considered the target PSM as false. The in silico digestion was performed in Python using the pyteomics.parser function from pyteomics with the following settings: Trypsin digestion, two allowed missed cleavages, minimum peptide length of six [6, 7].”*

*·	For bulk RNA-seq DE analysis, we have added another benchmark dataset and another way of generating synthetic data with ground truths. For the reviewer’s convenience, below we have pasted the paragraphs in Section “DEG identification from bulk RNA-seq data (differential analysis I)”.*

*“We compared Clipper with two popular DEG identification methods: edgeR [8] and DESeq2 [9] (Supp. Section S5.3). Specifically, when we implemented Clipper, we first performed the trimmed mean of M values (TMM) normalization [10] to correct for batch effects; then we treated genes as features and their normalized expression levels as measurements under two conditions (Supp. Section S7.3). We also implemented two versions of DESeq2 and edgeR: with or without IHW, a popular procedure for boosting the power of p-value-based FDR control methods by incorporating feature covariates [11]. In our implementation of the two versions of DESeq2 and edgeR, we used their standard pipelines, including normalization, model fitting, and gene filtering (edgeR only). To verify the FDR control, we generated four realistic synthetic datasets from two real RNA-seq datasets—one from classical and non¬classical human monocytes [12] and the other from yeasts with or without snf2 knockout [13]—using simulation strategies 1 and 2 (Supp. Section S6.3).*

*In detail, in simulation strategy 1, we used bulk RNA-seq samples from two conditions to compute a fold change for every gene between the two conditions; then we defined true DEGs as the genes whose fold changes exceeded a threshold; next, we randomly drew three RNA-seq samples and treated them as replicates from each condition (m = n = 3 as in Methods); using those subsampled replicates of two conditions, we preserved the true DEGs’ read counts and permuted the read counts of the true non-DEGs, i.e., the genes other than true DEGs, between conditions. In summary, simulation strategy 1 guarantees that the measurements of true non-DEGs are i.i.d., an assumption that Clipper relies on for theoretical FDR control.*

*In simulation strategy 2, borrowed from a benchmark study [14], we first randomly selected at most 30% genes as true DEGs; next, we randomly drew six RNA-seq samples from one condition (classical human monocytes and yeasts without knockout) and split the samples into two “synthetic conditions,” each with three replicates (m = n = 3 as in Methods); then for each true DEG, we multiplied its read counts under one of the two synthetic conditions (randomly picked independently for each gene) by a randomly generated fold change (see Supp. Section S6.3); finally, for the true non-DEGs, we preserved their read counts in the six samples. In summary, simulation strategy 2 preserves batch effects, if existent in real data, for the true non-DEGs (the majority of genes). As a result, the semi-synthetic data generated under strategy 2 may violate the Clipper assumption for theoretical FDR control and thus can help evaluate the robustness of Clipper on real data.*

*The four semi-synthetic datasets have ground truths (true DEGs and non-DEGs) to evaluate each DEG identification method’s FDR and power for a range of target FDR thresholds: q = 1%, 2%, .. . , 10%. Our results in Fig. 3a and Supp. Figs. S15a–S17a show that Clipper consistently controls the FDR and achieves high power on all four semi-synthetic datasets. In contrast, DESeq2 and edgeR cannot consistently control the FDR except for the yeast semi-synthetic dataset generated under simulation strategy 2. Given the fact that DESeq2 and edgeR do not consistently perform well on the three other semi-synthetic datasets, we hypothesize that their parametric distributional assumptions, if violated on real data, hinder valid FDR control, in line with our motivation for developing Clipper. By examining whether true non-DEGs’ p-values calculated by DESeq2 or edgeR follow the theoretical Uniform[0, 1] distribution, we find that the answer is no for many non-DEGs, as indicated by the small p-values (one per non-DEG) of uniformity tests (Supp. Fig. S30); this issue is more serious for DESeq2, consistent with the worse FDR control of DESeq2 (Fig. 4 and Supp. Figs. S15– S17). Furthermore, we observe that adding IHW to edgeR and DESeq2 has negligible effects on the four semi-synthetic datasets.”*

*Figure S30: Histograms of p-values (one p-value per non-DEG) that are obtained by testing whether each non-DEG’s p-values (output by DESeq2 or edgeR) follow a uniform distribution. For each real dataset (yeasts on the top and human monocytes on the bottom) and each simulation strategy (red for strategy 1 and blue for strategy 2), a histogram is plotted for DESeq2 (left) or edgeR (right); each p-value is calculated across 100 semi-synthetic datasets (excluding NA p-values). In each panel, more right skewed histograms are considered better.*

*·	For Hi-C data, we are not exactly sure what the reviewer meant by “spatial correlation,” and we interpret it as the phenomenon that closer regions have higher contact intensities. Supp. Fig. S29b shows that our semi-synthetic Hi-C data exhibit this phenomenon.*

*Figure S29b: In one randomly picked Hi-C semi-synthetic dataset, closer genomic regions tend to have higher contact intensities.*

Comment R1.4 The theoretical calculations for FDR control rely on having the signs of contrast scores of uninteresting features to be equally likely positive or negative (e.g., Lemma 1). This is an assumption that will be violated for enrichment analysis in ChIP-seq data as the background of the ChIP sample is different than the “input” control by construction. This will lead to input to be generally (normalization also has a role to play here) slightly larger than the ChIP background counterpart. It is possible that the theoretical result will hold with a modification, but as I have indicated in R1.3, application to genomic problems require more investigation.

*Answer to R1.4 We thank the reviewer for the suggestion. Actually, to have theoretical FDR control, we only need uninteresting features’ contrast scores to be equally or more likely negative than positive. Hence, this condition still holds for ChIP-seq data, where uninteresting regions’ contrast scores are more likely negative than positive as the reviewer said (because uninteresting regions are likely to have higher values in the control sample than the ChIP sample). In Lemma 1, we wrote “equally likely” to make the theoretical condition more intuitive. In our revision, we have added the following sentence to the end of Section 2.2:*

*“Notably, Lemma 1(a) can be relaxed as IP(83 = 1) ≤ IP(83 = −1) for all j ∈. Then Lemma 2 still holds, and so does Theorem 1, making Clipper still have theoretical FDR control.”*

*We have also added the following sentence to Section “Peak calling from ChIP-seq data (en-richment analysis I)” in Results.*

*“It is known that uninteresting regions tend to have larger read counts in the control sample than in the experimental (ChIP) sample, making them more likely to have negative contrast scores than positive ones. However, this phenmenon does not violate Clipper’s theoretical assumption (Lemma 1(a) in Supp. Section S2.2), which can be relaxed as we note in Methods.”*

Comment R1.5 Finally, if the RNA-seq analysis focused on differential expression in scRNA-seq (in addition to bulk), the relevance of the paper for the genomics community would be higher.

*Answer to R1.5 We thank the reviewer for this valuable suggestion. We totally agree that single-cell RNA-seq (scRNA-seq) data can be an important application of Clipper. Following this, we have added a new application of Clipper: DEG identification from scRNA-seq data. We have added the results to Section “DEG identification from single-cell RNA-seq data (differential analysis II)” along with Fig. 4d (Page 18 of this response letter) and Supp. Fig. S16. For the reviewer’s convenience, below we have pasted the newly added text and Supp. Fig. S16:*

*“Single-cell RNA sequencing (scRNA-seq) technologies have revolutionized biomedical sciences by enabling genome-wide profiling of gene expression levels at an unprecedented single-cell reso¬lution. DEG analysis is widely applied to scRNA-seq data for discovering genes whose expression levels change between two conditions or between two cell types. Compared with bulk RNA-seq data, scRNA-seq data have many more “replicates” (i.e., cells, whose number is often in hundreds) under each condition or within each cell type.*

*We compared Clipper with edgeR [8], MAST [15], Monocle3 [16], the two-sample t test, and the Wilcoxon rank-sum test, five methods that are either popular or reported to have compara¬tively top performance from a previous benchmark study [17]. To verify the FDR control, we used scDesign2, a flexible probabilistic simulator to generate scRNA-seq count data with known true DEGs [18]. scDesign2 offers three key advantages that enable the generation of realistic synthetic scRNA-seq count data: (1) it captures distinct marginal distributions of different genes; (2) it preserves gene-gene correlations; (3) it adapts to various scRNA-seq protocols. Using scDesign2, we generated two synthetic scRNA-seq datasets from two real scRNA-seq datasets of peripheral blood mononuclear cells (PBMCs) [19]: one using 10x Genomics [20] and the other using Drop-seq [21]. Each synthetic dataset contains two cell types, CD4+ T cells and cytotoxic T cells, which we treated as two conditions. Having true DEGs known, the synthetic datasets allow us to evaluate Clipper’s and the other five methods’ FDRs and power for a range of target FDR thresholds: q = 1%, 2%, . . . , 10%. Fig. 4d and Supp. Fig. S16 show that on both 10x Genomics and Drop-seq synthetic datasets, Clipper consistently controls the FDR and remains the most powerful among all the methods that achieve FDR control. These results demonstrate Clipper’s robust performance in scRNA-seq DEG analysis.”*

*Figure S16: Comparison of Clipper and five scRNA-seq DEG identification methods on synthetic Drop-seq data generated by scDesign2 (based on a real Drop-seq dataset of PBMCs). The target FDR threhold ranges from 1% to 10%. In the “Actual FDR vs. Target FDR” plot (left), points above the dashed diagonal line indicate failed FDR control. Clipper controls the FDR while maintaining high power, demonstrating Clipper’s good performance in single-cell DE analyses*

Reviewer 2

This is an interesting, potentially impactful, contribution to an important aspect of genomic data analysis. The authors describe new methodology to more faithfully control for False Discov¬ery Rate than currently established methodology. The proposed methodology adapts previous work on selection procedures from Candes to testing situations applicable across multiple genomic measurements. Overall, the metodological contribution is an innovative application and is well characterized formally (using proof mechanisms as established in the Candes line of work). The empirical characterization of the method as provided here can be improved.

*We thank the reviewer for the comments, and we have added more validation and comparison to our revised manuscript. Please see our point-by-point response below.*

Main comments:

Comment R2.1 Contrast Scores. The statistics on which this method relies are contrast scores, of which the authors present two, one based on differences between empirical means of two groups (diff), and one based on a signed maximum absolute value of means of two groups (enrich).

*Answer to R2.1 We would like to clarify that our two contrast scores were referred to as the difference contrast score (“diff”) and the maximum contrast score (“max”), which are not the same as the differential analysis and the enrichment analysis, respectively. In other words, the difference and maximum contrast scores do not have a one-to-one correspondence to the differential and enrichment analyses; both contrast scores can be used for both analyses. Realizing that the contrast score names might be confusing to readers (as “diff” appears in both “difference contrast score” and “differential analysis”), we have renamed “difference contrast score” as “minus contrast score.” For the reviewer’s convenience, we have pasted below the definitions of the summary statistics and contrast scores for the enrichment analysis with m = n (i.e., two conditions have the same number of replicates). For other analyses, the minus and maximum contrast scores are defined in another way (Page 13 in Methods of the revised manuscript).*

*“To construct contrast scores, Clipper uses two summary statistics t(•,•) : Rm≥0 Rn≥0 -+ R to extract data information regarding whether a feature is interesting or not:*

I find the introduction of the enrich contrast score, confusing and perhaps unnecessary. As far as I could understand, the latter is mainly applicable in the enrichment testing case (average of one group is strictly greater than the other group), but the diff contrast score is also applicable in that case. And it seems that empirically the diff contrast score has better performance as well. That is, there is no evidence, as far as I could tell, of the enrichment contrast score providing a practical or theoretical advantage.

*Answer to R2.1 We would like to clarify that there is no “enrichment contrast score,” and we assume that the reviewer meant the “maximum contrast score” in our following reply.*

I would recommend

(a)	providing evidence of this practical advantage, or simplify the presentation of the paper by considering only the diff contrast score.

(b)	guidelines on specific situations when each kind of contrast score is appropriate. For instance when testing the Clipper R tool and using the DEG example, the ”analysis” parameter is set to ”differential” but the contrast score used by default is ”max”, which I found surprising. In general, I find the whole need for this second type of contrast score confusing

*Answer to R2.1 We thank the reviewer for these suggestions, and we apologize for the confusion caused by the nomenclature in our previous version. In fact, Clipper uses the minus contrast score (“minus”) in enrichment analysis with m = n and the maximum contrast score (“maximum”) in other analyses. This choice of contrast score is guided by our extensive power comparison results in simulation. We plan to investigate the power difference of these two contrast scores and other alternatives in a future study. To clarify our choice of contrast score, we have added the following sentences to Methods (Page 16 of the revised manuscript).*

*“For both differential and enrichment analyses, the two contrast scores (minus and maximum) can both control the FDR. Based on the power comparison results in Supp. Section S3 and Supp. Figs. S22–S25, Clipper has the following default choice of contrast score: for the enrichment anal¬ysis when two conditions have the same number of replicates (“Enrichment analysis with equal numbers of replicates (m = n)” in Methods), Clipper uses the BC procedure with the minus con¬trast score; for the enrichment analysis when two conditions have different numbers of replicates (“Enrichment analysis with any numbers of replicates m and n” in Methods) or the differential analysis (“Differential analysis with m + n > 2” in Methods), Clipper uses the GZ procedure with maximum contrast score.”*

Comment R2.2 Applicability beyond two-group testing. The limitation of this procedure to two-group testing is a severe handicap. In a majority of analysis we tend to see the need to include additional terms in, e.g., linear regression models to control for technical or biological sources of variability beyond the direct effect being tested. Given that the original application from Candes et al. was selection in linear models, wouldn’t using parameter estimates for appropriately setup up covariates (related to the diff contrast score) as contrast scores work here as well? The authors should comment on this on the manuscript, if extending the method to this is beyond scope of the paper.

*Answer to R2.2 We thank the reviewer for this valuable suggestion. We indeed think that Clipper can be extended to the regression framework so that the test can be done by adjusting for other covariates. Accordingly, we have added the following text to the Discussion section to discuss the extension of Clipper beyond two-group testing.*

*“Our current version of Clipper allows the identification of interesting features between two conditions. However, there is a growing need to generalize our framework to identify features across more than two conditions. For example, temporal analysis based on scRNA-seq data aims to identify genes whose expression levels change along time [22]. To tailor Clipper for such analysis, we could define a new contrast score that differentiates the genes with stationary expression (un¬interesting features) from the other genes with varying expression (interesting features). Further studies are needed to explore the possibility of extending Clipper to the regression framework so that Clipper can accommodate data of multiple conditions or even continuous conditions, as well as adjusting for confounding covariates.”*

*We have also added the following sentences to the Discussion section to discuss the contrast scores that utilize linear regression models to control for technical or biological sources of variability.*

*“Beyond our current interpretation, however, we admit that future studies are needed to explore alternative contrast scores and their power with respect to data characteristics and analysis tasks. Furthermore, we may generalize Clipper to be robust against sample batch effects by constructing the contrast score as a regression-based test statistic that has batch effects removed.”*

Comment R2.3 Simulations. I am not sure the simulations are designed to be the most infor¬mative. I am concerned that the bulk of the simulations are focused on cases with very small number of replicates (1 v. 1, 3 v. 3). This is becoming increasingly uncommon (perhaps not in the proteomics and hi-c use cases?) and would prefer that simulations are refocused to concentrate on what may be more important characteristics:

(a)	behavior for moderate sample sizes (this is included as 10v10 testing but not highlighted, I suspect because the difference in performance to other methods is not as significant),

(b)	spends more time discussing how the performance of these methods degrade as the indepen-dence assumption across tests is violated (this is a pervasive problem in genomic analysis and it is cursorily addressed in the current set of simulations. In particular, there is no case where correlated tests are used in Poisson or Negative Binomial tests, only on the Normal case.

(c)	In many of the plots there is no distincation made of the data generating distribution (between Normal, Poisson or NB), and is hard to understand behavior of the methods.

(d)	the simulations concentrate on situations where variability across tests is fairly stable whereas (for instance in the NB case all features have the same overdispersion parameter). This is not reflective of many genomic measurements where there is a fair amount of differences in variation across features.

*Answer to R2.3 We thank the reviewer for the detailed suggestions. First, as the reviewer said with a question mark (“(perhaps not in the proteomics and hi-c use cases?)”), indeed, the numbers of replicates are still small for most proteomics and Hi-C data. In our previous version, we focused on enrichment and differential analyses with small numbers of replicates because that is the scenario where parametric tests have questionable p-values (as parametric assumptions can never be verified with such small numbers of replicates) and non-parametric tests do not have good power.*

*Second, following the reviewer’s suggestions, we have added the following analysis.*

*(a) We have modified Supp. Fig. S10 (pasted below for the reviewer’s convenience)—the 10vs10 enrichment analysis—by adding (1) settings with 10% outlier samples (panel b) and (2) additional generic FDR control methods for benchmarking Clipper. The results show that Clipper consistently controls the FDR for three distributional families (Gaussian, Poisson, and negative binomial), regardless of the existence of outliers. Clipper also achieves the highest power among all the methods that control the FDR. Overall, the conclusions from this 10vs10 enrichment analysis are consistent with those from the 3vs3 enrichment analysis.*

*We have also added a new application of Clipper: DEG identification from scRNA-seq data. In typical scRNA-seq DEG analysis, the numbers of replicates (i.e., cells) under the two conditions are in the hundreds (e.g., 1172 and 789 in the Drop-seq dataset; 963 and 694 in the 10x Genomics dataset). Since Clipper achieves valid FDR control and the highest power in our scRNA-seq data analysis, we demonstrate that Clipper performs well for large sample sizes. We have added the results to Section “DEG identification from single-cell RNA-seq data (differential analysis II)” along with Fig. 4d and Supp. Fig. S16. For the reviewer’s convenience, we have pasted the newly added text and figures in our response to Reviewer 1’s Comment 1.5 (Pages 23–24 in this response letter).*

*Figure S10: In the 10vs10 enrichment analysis on data (a) without or (b) with outliers, comparison of Clipper and eight generic FDR control methods (BH-pooled, BH-pair-Wilcoxon, BH-pair-parametric, and BH-pair-permutation, BH-pair-2as1, BH-pair-mis, locfdr-emp, and locfdr-swap) in terms of their FDR control and power. At target FDR thresholds ∈1% 2%--- 10%, each method’s actual FDRs and power are evaluated on 200 simulated datasets with = 10000 features generated from the Gaussian distribution, the Poisson distribution, or the negative binomial distribution under heterogeneous back-ground scenarios. Clipper achieves the highest power for all three distributions regardless of the existence of outliers.*

*(b)	Although we only included feature correlations in our simulation under the Gaussian setting, we have added two real data applications to show the performance of Clipper in the presence of feature correlations. First, in the above scRNA-seq application, synthetic data are gen-erated with gene correlations by scDesign2, a probabilistic simulator that generates realistic scRNA-seq data by preserving gene correlations in real data; marginal distributions of genes under each condition are negative binomial distributions in the synthetic data. Second, in the bulk RNA-seq application, features are genes that have biological correlations, and each gene has a discrete count distribution. The good performance of Clipper in these two appli¬cations shows that Clipper is likely robust to the violation of the independence assumption in real data analysis.*

*(c)	We do not exactly understand what the reviewer meant by “no distinction made of the data generating distribution.” By checking our plots carefully, we found that we labeled the data generating distribution in every figure panel except Supp. Figs. S9 and S11. In our revision, we have added explicit labels “Negative binomial” and “Gaussian” to these two figures, respectively (the two figures are pasted below for the reviewer’s convenience). Moreover, we would like to clarify that we included three data generating distributions in simulation to show that Clipper is a versatile method working for multiple common distributions. Hence, we actually expect Clipper to have similar advantages over parametric tests when the numbers of replicates are small, regardless of data generating distributions. To avoid confusion, we have added the following sentence to the section “Clipper has verified FDR control and power advantage in simulation” (Page 5 in the revised manuscript):*

*“Moreover, the above five phenomena are consistently observed across the three distributions (Gaussian, Poission, and negative binomial) that we have examined, further confirming the robustness of Clipper.”*

*(d)	To address this issue that our previous simulations have fairly stable variability of features, we have added a new simulation study and a new real data application. In the new simulation study, we generated features from Gaussian distributions with varying variances (Supp. Sec-tion S3), and we show that Clipper still has robust performance (Supp. Fig. S13; pasted on Page 6 in this response letter). The newly added real data application is the aforementioned scRNA-seq DEG analysis (Pages 23–24 of this response letter), where genes have negative binomial parameters estimated from real data, and Clipper still has good performance (Fig. 4c on Page 18 and Supp. Fig. S16 on Page 24 in this response letter).*

*Figure S9: In the 3vs3 differential analysis without (columns 1 and 3) or with outliers (columns 2 and 4), comparison of Clipper, BH-pair-correct (known dispersion), and BH-pair-correct (unknown dispersion) in terms of their FDR control and power. At target FDR thresholds ∈1% 2%--- 10%, each method’s actual FDRs and power are evaluated on 200 simulated datasets with = 10000 features generated from the negative binomial distribution under homogeneous (two left columns) and heterogeneous (two right columns) background scenarios. BH-pair-correct (unknown dispersion) cannot control the FDR in all settings. In contrast, Clipper is consistently the most powerful for homogeneous and heterogeneous background.*

*Figure S11: In the 3vs3 enrichment analysis with correlated features, comparison of Clipper and six other generic FDR control methods (BH-pooled, BH-pair-correct, BH-pair-2as1, BH-pair-mis, locfdr-emp, and locfdr-swap) in terms of their FDR control and power in 3vs3 enrichment analysis. At target FDR thresholds ∈1% 2%••• 10%, each method’s actual FDRs and power are evaluated on 200 simulated datasets with = 10000 features generated from a multivariate Gaussian distribution with a correlation 02 (columns 1 and 3) or 04 (columns 2 and 4) between features. Among the methods that control the FDR, Clipper is the second most powerful for homogeneous background and the most powerful for heterogeneous background*

Comment R2.4 differential expression analysis. I am sincerely surprised that there is such a difference in behavior between DESeq2 and EdgeR in this analysis. It would be good to better understand how these methods were applied, for instance, I would expect that the regularization method approach in DESeq2 would result in lower number of significant results. I did not see specifics of how these methods were used in the paper. Similarily, in my experience for common RNA-seq datasets using importance hypothesis weighting tends to have moderate to substantial effects, yet I am surprised that it had no effect in this case. I would suggest, (a) that the authors provide more detail on how these methods were used, and that (b) an additional analysis is included, ideally using an established benchmark dataset.

*Answer to R2.4 We thank the reviewer for these detailed suggestions. Following them, we have added the following detailed explanation and a new benchmark analysis.*

*·	We implemented both DESeq2 and edgeR using the standard pipelines suggested by their tutorials.*

*– For DESeq2, we first used DESeqDataSetFromMatrix to generate a DESeqDataSet ob¬ject. Then we fitted the model using DESeq. Finally, we extracted p-values using the function results() with the parameter alpha (specifying the target FDR threshold) set to 0.99999 so that we could obtain all genes’ p-values. To implement IHW, we first computed a covariate for each genes as its average read count across all samples under the two conditions. We then applied IHW using the function ihw with the parameter alpha (specifying the target FDR threshold) set to q, our target FDR threshold and the parameter covariates set to our computed covariate.*

*– For edgeR, we first created a DGEList object and filtered genes using the function filterByExpr. Then we estimated the negative binomial dispersion using the function estimateDisp and then fit the negative binomial model using quasi-likelihood by the function glmQLFit. Finally, we extracted p-values using the function glmQLFTest. We implemented IHW in the same way as for DESeq2.*

*·	Following the reviewer’s advice and Reviewer 1’s suggestions, we have added another bench-mark dataset and another way of generating synthetic data with ground truths. For the reviewer’s convenience, below we have pasted the paragraphs in Section “DEG identification from bulk RNA-seq data (differential analysis I)”. Fig. 3 and Supp. Figs. S15–S17 are on Pages 10–13 in this response letter.*

*“We compared Clipper with two popular DEG identification methods: edgeR [8] and DESeq2 33 [9] (Supp. Section S5.3). Specifically, when we implemented Clipper, we first performed the trimmed mean of M values (TMM) normalization [10] to correct for batch effects; then we treated genes as features and their normalized expression levels as measurements under two conditions (Supp. Section S7.3). We also implemented two versions of DESeq2 and edgeR: with or without IHW, a popular procedure for boosting the power of p-value-based FDR control methods by incorporating feature covariates [11]. In our implementation of the two versions of DESeq2 and edgeR, we used their standard pipelines, including normalization, model fitting, and gene filtering (edgeR only). To verify the FDR control, we generated four realistic synthetic datasets from two real RNA-seq datasets—one from classical and non¬classical human monocytes [12] and the other from yeasts with or without snf2 knockout [13]—using simulation strategies 1 and 2 (Supp. Section S6.3).*

*In detail, in simulation strategy 1, we used bulk RNA-seq samples from two conditions to compute a fold change for every gene between the two conditions; then we defined true DEGs as the genes whose fold changes exceeded a threshold; next, we randomly drew three RNA-seq samples and treated them as replicates from each condition (m = n = 3 as in Methods); using those subsampled replicates of two conditions, we preserved the true DEGs’ read counts and permuted the read counts of the true non-DEGs, i.e., the genes other than true DEGs, between conditions. In summary, simulation strategy 1 guarantees that the measurements of true non-DEGs are i.i.d., an assumption that Clipper relies on for theoretical FDR control.*

*In simulation strategy 2, borrowed from a benchmark study [14], we first randomly selected at most 30% genes as true DEGs; next, we randomly drew six RNA-seq samples from one condition (classical human monocytes and yeasts without knockout) and split the samples into two “synthetic conditions,” each with three replicates (m = n = 3 as in Methods); then for each true DEG, we multiplied its read counts under one of the two synthetic conditions (randomly picked independently for each gene) by a randomly generated fold change (see Supp. Section S6.3); finally, for the true non-DEGs, we preserved their read counts in the six samples. In summary, simulation strategy 2 preserves batch effects, if existent in real data, for the true non-DEGs (the majority of genes). As a result, the semi-synthetic data generated under strategy 2 may violate the Clipper assumption for theoretical FDR control and thus can help evaluate the robustness of Clipper on real data.*

*The four semi-synthetic datasets have ground truths (true DEGs and non-DEGs) to evalu¬ate each DEG identification method’s FDR and power for a range of target FDR thresholds: q = 1%, 2%, .. . , 10%. Our results in Fig. 3a and Supp. Figs. S15a–S17a show that Clipper consistently controls the FDR and achieves high power on all four semi-synthetic datasets. In contrast, DESeq2 and edgeR cannot consistently control the FDR except for the yeast semi-synthetic dataset generated under simulation strategy 2. Given the fact that DESeq2 and edgeR do not consistently perform well on the three other semi-synthetic datasets, we hypothesize that their parametric distributional assumptions, if violated on real data, hinder valid FDR control, in line with our motivation for developing Clipper. By examining whether true non-DEGs’ p-values calculated by DESeq2 or edgeR follow the theoretical Uniform[0, 1] distribution, we find that the answer is no for many non-DEGs, as indicated by the small p-values (one per non-DEG) of uniformity tests (Supp. Fig. S30); this issue is more serious for DESeq2, consistent with the worse FDR control of DESeq2 (Fig. 4 and Supp. Figs. S15– S17). Furthermore, we observe that adding IHW to edgeR and DESeq2 has negligible effects on the four semi-synthetic datasets.”*

*·	Moverover, we did literature search and found abundant evidence showing that DESeq2 and edgeR performed differently on real data [23].*

*References*

*[1]	R. F. Barber and E. J. Candès, “Controlling the false discovery rate via knockoffs,” The Annals of Statistics, vol. 43, no. 5, pp. 2055–2085, 2015.*

*[2]	J. R. Gimenez and J. Zou, “Improving the stability of the knockoff procedure: Multiple simultaneous knockoffs and entropy maximization,” arXiv preprint arXiv:1810.11378, 2018.*

*[3]	Q. Li, J. B. Brown, H. Huang, and P. J. Bickel, “Measuring reproducibility of high-throughput experiments,” The annals of applied statistics, vol. 5, no. 3, pp. 1752–1779, 2011.*

*[4]	D. Mellacheruvu, Z. Wright, A. L. Couzens, J.-P. Lambert, N. A. St-Denis, T. Li, Y. V. Miteva, S. Hauri, M. E. Sardiu, T. Y. Low, et al., “The crapome: a contaminant repository for affinity purification–mass spectrometry data,” Nature methods, vol. 10, no. 8, pp. 730–736, 2013.*

*[5]	J. Cox and M. Mann, “Maxquant enables high peptide identification rates, individualized ppb-range mass accuracies and proteome-wide protein quantification,” Nature biotechnology, vol. 26, no. 12, pp. 1367–1372, 2008.*

*[6]	A. A. Goloborodko, L. I. Levitsky, M. V. Ivanov, and M. V. Gorshkov, “Pyteomics—a python framework for exploratory data analysis and rapid software prototyping in proteomics,” Jour¬nal of The American Society for Mass Spectrometry, vol. 24, no. 2, pp. 301–304, 2013.*

*[7]	L. I. Levitsky, J. A. Klein, M. V. Ivanov, and M. V. Gorshkov, “Pyteomics 4.0: five years of development of a python proteomics framework,” Journal of proteome research, vol. 18, no. 2, pp. 709–714, 2018.*

*[8]	M. D. Robinson, D. J. McCarthy, and G. K. Smyth, “edger: a bioconductor package for differential expression analysis of digital gene expression data,” Bioinformatics, vol. 26, no. 1, pp. 139–140, 2010.*

*[9]	M. I. Love, W. Huber, and S. Anders, “Moderated estimation of fold change and dispersion for rna-seq data with deseq2,” Genome biology, vol. 15, no. 12, p. 550, 2014.*

*[10]	M. D. Robinson and A. Oshlack, “A scaling normalization method for differential expression analysis of rna-seq data,” Genome biology, vol. 11, no. 3, pp. 1–9, 2010.*

*[11]	N. Ignatiadis, B. Klaus, J. B. Zaugg, and W. Huber, “Data-driven hypothesis weighting increases detection power in genome-scale multiple testing,” Nature methods, vol. 13, no. 7, pp. 577–580, 2016.*

*[12]	C. R. Williams, A. Baccarella, J. Z. Parrish, and C. C. Kim, “Empirical assessment of anal¬ysis workflows for differential expression analysis of human samples using rna-seq,” BMC bioinformatics, vol. 18, no. 1, p. 38, 2017.*

*[13]	M. Gierli´nski, C. Cole, P. Schofield, N. J. Schurch, A. Sherstnev, V. Singh, N. Wrobel, K. Gharbi, G. Simpson, T. Owen-Hughes, et al., “Statistical models for rna-seq data derived from a two-condition 48-replicate experiment,” Bioinformatics, vol. 31, no. 22, pp. 3625–3630, 2015.*

*[14]	K. Korthauer, P. K. Kimes, C. Duvallet, A. Reyes, A. Subramanian, M. Teng, C. Shukla, E. J. Alm, and S. C. Hicks, “A practical guide to methods controlling false discoveries in computational biology,” Genome biology, vol. 20, no. 1, pp. 1–21, 2019.*

*[15]	G. Finak, A. McDavid, M. Yajima, J. Deng, V. Gersuk, A. K. Shalek, C. K. Slichter, H. W. Miller, M. J. McElrath, M. Prlic, et al., “Mast: a flexible statistical framework for assessing transcriptional changes and characterizing heterogeneity in single-cell rna sequencing data,” Genome biology, vol. 16, no. 1, pp. 1–13, 2015.*

*[16]	X. Qiu, A. Hill, J. Packer, D. Lin, Y.-A. Ma, and C. Trapnell, “Single-cell mrna quantification and differential analysis with census,” Nature methods, vol. 14, no. 3, pp. 309–315, 2017.*

*[17]	C. Soneson and M. D. Robinson, “Bias, robustness and scalability in single-cell differential expression analysis,” Nature methods, vol. 15, no. 4, p. 255, 2018.*

*[18]	T. Sun, D. Song, W. V. Li, and J. J. Li, “scdesign2: an interpretable simulator that generates high-fidelity single-cell gene expression count data with gene correlations captured,” bioRxiv, 2020.*

*[19]	J. Ding, X. Adiconis, S. K. Simmons, M. S. Kowalczyk, C. C. Hession, N. D. Marjanovic, T. K. Hughes, M. H. Wadsworth, T. Burks, L. T. Nguyen, et al., “Systematic comparison of single-cell and single-nucleus rna-sequencing methods,” Nature biotechnology, vol. 38, no. 6, pp. 737–746, 2020.*

*[20]	G. X. Zheng, J. M. Terry, P. Belgrader, P. Ryvkin, Z. W. Bent, R. Wilson, S. B. Ziraldo, T. D. Wheeler, G. P. McDermott, J. Zhu, et al., “Massively parallel digital transcriptional profiling of single cells,” Nature communications, vol. 8, no. 1, pp. 1–12, 2017.*

*[21]	E. Z. Macosko, A. Basu, R. Satija, J. Nemesh, K. Shekhar, M. Goldman, I. Tirosh, A. R. Bialas, N. Kamitaki, E. M. Martersteck, et al., “Highly parallel genome-wide expression pro¬filing of individual cells using nanoliter droplets,” Cell, vol. 161, no. 5, pp. 1202–1214, 2015.*

*[22]	D. Song and J. J. Li, “Pseudotimede: inference of differential gene expression along cell pseudotime with well-calibrated p-values from single-cell rna sequencing data,” bioRxiv, 2020.*

*[23]	B. Baik, S. Yoon, and D. Nam, “Benchmarking rna-seq differential expression analysis meth¬ods using spike-in and simulation data,” PloS one, vol. 15, no. 4, p. e0232271, 2020.*

**Second round of review**

**Reviewer 1**

The authors did an excellent job of carrying out more computational experiments and providing ample discussion for clarifying several key points. All of my comments were satisfactorily addressed.
